# Dance/movement therapy for improving balance ability and bone mineral density in long-term patients with schizophrenia: a randomized controlled trial

**DOI:** 10.1038/s41537-023-00373-w

**Published:** 2023-07-31

**Authors:** Hengyong Guan, Zhaoxia Zhou, Xipo Li, Yanfen Pan, Zhenmin Zou, Xiangfei Meng, Kunya Guan, Lie Zhang, Zhanmin Li, Xueling Li, Baochun Wei, Xuan Zhang, Weiqing Li, Dongmei Han, Zezhi Li, Meihong Xiu

**Affiliations:** 1 Hebei Province Veterans Hospital, Hebei, China; 2 Department of Nutritional and Metabolic Psychiatry, Affiliated Brain Hospital of Guangzhou Medical University, Guangzhou, China; 3 Guangdong Engineering Technology Research Center for Translational Medicine of Mental Disorders, Guangzhou, China; 4 Key Laboratory of Neurogenetics and Channelopathies of Guangdong Province and the Ministry of Education of China Guangzhou Medical University, Guangzhou, China; 5 Peking University HuiLongGuan Clinical Medical School, Beijing HuiLongGuan Hospital, Beijing, China

**Keywords:** Schizophrenia, Psychiatric disorders

## Abstract

Fractures are common accidents for long-term hospitalized patients with schizophrenia (SZ) in psychiatric hospitals, and once they occur, patients usually endure the pain of fractures for a long time. Accumulating evidence has supported the implementation of dance/movement therapy (DMT) as a promising intervention for patients with SZ. However, no research has been conducted to investigate its role in balance ability in SZ. This study was designed to investigate the efficacy of a 12-week DMT intervention in bone mineral density and balance ability in patients with SZ using a randomized, controlled trial design. A total of 58 veterans with SZ were randomly assigned to the DMT intervention group (*n* = 29) and the treatment-as-usual (TAU) group (*n* = 29). Bone mineral density (BMD) and balance ability were measured in both groups at two measurement points (at baseline and at the end of Week 12). We found that patients in the DMT intervention group had significant improvements in BMD and balance ability compared with the TAU group by using repeated measures analysis of variance. Treatment with DMT demonstrated a significant improvement in BMD from baseline to week 12 (0.03, 95% CI: 0.01–0.05). For the Berg total score and static and dynamic balance, the mean changes in the DMT group were 7.3 (95% CI: 5.6–9.0), 4.0 (95% CI: 0.9–7.1), and 3.7 (95% CI: 2.6–4.8), respectively. Regression analysis showed that baseline BMD was a significant predictor of improvement in BMD from baseline to week 12 in the DMT group (*β* = 0.58, *p* < 0.001). Our results suggest for the first time that DMT intervention may be effective in beneficially regulating BMD and balance ability in SZ patients.

## Introduction

Schizophrenia (SZ) is a chronic and severe psychiatric disorder that affects approximately 1% of the population^1^. The psychiatric symptoms and high rate of relapse of SZ can significantly impair cognitive and social functioning, including problem-solving skills, interpersonal relationships, and work performance^2,3^. Moreover, individuals with SZ usually have higher disability and premature mortality rates compared to the general population^4^.

Fall is a common concern in hospital settings, with studies reporting a fall rate between three and five per 1000 bed days^5–7^. A previous meta-analysis showed that the pooled rate of fractures per 1000 person-years was 5.54 in SZ, compared to 3.48 in the general population^8^. Between 1% and 3% of falls in hospitals result in a fracture, and even minor injuries can cause distress and delay rehabilitation^9,10^. Osteoporosis and its precursors, e.g., osteopenia and low bone mineral density (BMD), have been reported in chronic patients with SZ^11–14^, reviewed in Kishimoto et al.^12^. They were strong predictors for subsequent falls and fractures in psychiatric hospitals^8,15^. There is evidence that nearly 52% of patients with SZ have low BMD, 13.2% of patients with osteoporosis, and 40.0% of patients with osteopenia^14,16^. Studies have demonstrated that older age, male gender, unhealthy lifestyle factors, increased levels of stress hormones, diabetes, and hypertension were associated with an increased risk of osteoporosis^8,17^. Among the risk factors, lower limb muscle strength and balance ability are significant predictors of fractures and falls, which are known to be impaired in patients with SZ^18,19^. Considering a deficit in general preventative medical care for osteoporosis in individuals with SZ, specialized exercises provided by specialists such as exercise physiotherapists may play a key role in the management of low BMD in SZ patients^20^. Therefore, further studies are warranted to investigate and elucidate the role of exercise in improving limb strength and preventing falls in long-term hospitalized patients.

Dance/movement therapy (DMT), as one of the creative arts therapies^21^, is an emerging therapy for the rehabilitation of patients with SZ in recent years^22–24^. DMT therapy uses movement, dance, and interpersonal communication to explore emotional, cognitive, social, and physical integration, enabling patients to enhance self-expression, accept and reconnect with their bodies, and strengthen their fitness^25^. Patients share their emotions, concerns, and coping strategies with others through dance/movement^26,27^. In particular, a few studies have reported a critical role of DMT intervention in negative symptoms in chronic patients with SZ^28,29^. However, there is still a lack of evidence on whether DMT is an effective therapy to increase the mean BMD and reduce the risk of fractures due to falls in long-term hospitalized patients with SZ.

Antipsychotic medications are currently effective in treating the symptoms of SZ, but antipsychotics can also cause adverse effects such as weight, muscle stiffness, and low BMD loss^30–36^. Although the etiology of low BMD in patients with SZ is complicated, studies have suggested that increased prolactin levels caused by the dopamine D_2_ receptor-blocking effect of antipsychotics can accelerate bone loss and increase the risk of osteoporosis^37^. Therefore, the risk of unintentional falls increases with long hospitalizations and long-term antipsychotic use in SZ patients^38^. Decreased BDM, changes in body composition, and skeletal muscle mass index were reported in elderly chronic SZ patients on long-term antipsychotic medications^11^. In the present study, we hypothesized that the DMT intervention was effective in increasing BMD and balance ability in long-term hospitalized male veterans with SZ as compared to the control group.

## Methods and materials

### Participants

Eighty long-term hospitalized male veterans with chronic SZ were recruited from Hebei Province Veterans Hospital through advertisements in this study. An experienced psychiatrist performed eligibility screening. The inclusion criteria included: (1) a diagnosis of SZ using the DSM-V; (2) male veterans aged 40–60 years old with a disease duration of 5 years or more and a long-term hospitalization of 3 years or more; (3) able to understand Mandarin Chinese; (4) legally eligible to sign an informed consent form; (5) stability of current antipsychotic medications for more than 2 years; (6) reduced bone mass, osteoporosis, and severe osteopenia after bone density analysis; (7) no comorbid serious physical illness and physical impairments and able to cooperate with nurses to complete the general intensity of activity training; and (8) consent for pre- and post-session interviews. The exclusion included: (1) substance dependence or abuse; (2) hypercalcemia and hyperuricemia; (3) history of kidney stones or renal calculi; and (4) lower limb injury and motor dysfunction, and inability to complete DMT intervention for various reasons. These situations are often associated with the inability to complete the therapy and dropout, and could thus lead to unequal sample sizes and biased results and threaten the validity of the results. The study protocol was approved by the institutional review board of Hebei Province Veterans Hospital. Written informed consent was obtained from each participant.

**Fig. 1:**
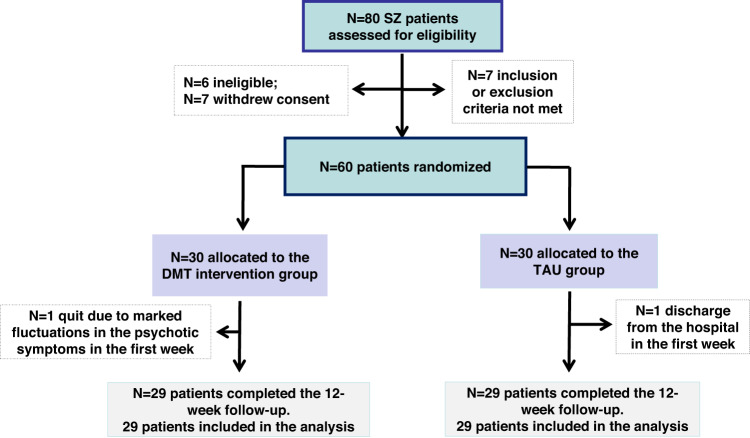
CONSORT flow diagram.

Of the 80 patients, 6 patients were ineligible, 7 patients did not meet the inclusion/exclusion criteria, 7 patients withdrew consent, and finally, 60 patients were randomly assigned. Thirty patients were assigned to the intervention group, and 30 patients were assigned to the treatment-as-usual (TAU) group. However, 1 participant in the TAU group dropped out of this study due to discharge from the hospital in the first week of treatment, and 1 patient in the DMT group was unable to complete DMT therapy due to marked fluctuations in the psychotic symptoms of SZ. Therefore, we included 58 patients (29 patients in each group) in the following analyses (Fig. 1).

Sample size power was calculated based on expected changes in BMD, which was the primary outcome of this study. The sample size is considered to achieve significance with a moderate effect size (ES) (*d* = 0.30), a power of 80%, and *α* = 0.05. A flowchart of the present trial is displayed in Fig. 1.

### Randomization and masking

This was a randomized, blinded, controlled clinical trial. There was no blinding due to the experience of therapists in applying the intervention. Outcome evaluators and patients remained blinded during the 12 weeks of treatment. However, patients sometimes shared their intervention experience, which may lead to the break of blinding. Eligible patients were randomly allocated (1:1) to receive either DMT intervention or TAU based on a computer-generated sequence. The independent third party, who was blinded to the subject groups, divided the patients into two groups.

### Interventions

The clinical trial used a two-arm parallel group design to compare TAU and DMT plus TAU on outcome measures at two time points.

### DMT Intervention

The group consisted of a dance movement therapist and 30 hospitalized veterans with SZ who chose to participate in this study. The DMT intervention was given twice a week for one hour each time. All patients received 24 sessions of DMT over a period of three months. DMT intervention was conducted by an experienced dance/movement therapist with over 10 years of experience in DMT interventions. The DMT intervention protocol was developed based on the therapist’s experience and the theoretical frameworks, and the DMT approach was described in a previous study^39^. In brief, the therapist used a body-based approach that included movement warm-ups followed by an improvisational dance. It starts with simple warm-up exercises such as breathing and muscle relaxation and continues with body awareness exercises. First, meeting, warming up, determining the group rules, explaining the principles, and determining the expectations activities. Then, the greeting is completed with warm-up, initiation, continuation, and closing activities. During the warm-up phase, the whole group comes together to form a circle, generally standing, in order to ensure the group dynamic. The detailed process of DMT intervention was described in the Supplementary file.

### Treatment as usual

The patients in the control group did not participate in the DMT intervention, but they were treated as usual. The TAU group sessions were conducted by licensed professionals employed at the hospital. The patients received regular daily antipsychotic medication and supportive psychotherapy once a week. Supportive psychotherapy was applied to the treatment of the patients once a week as a total of 12 sessions of 45 min over 3 months. They also participated in daily activities from Monday to Friday for at least 2 h each day, including indoor activities (watching TV, playing games, playing poker) and outdoor activities (walking and doing radio gymnastics).

### BMD measurement in patients

The primary outcome measure was the BMD of the patients recruited, which was assayed by an Erik AKDX-09W-1 bone density device (Shenzhen Ekere Electric Co.). The test was performed using the dual-energy X-ray absorptiometry method. Measurements were made at the distal end of the flexor bone of the patient’s left forearm for fan beam and line scan, which were reviewed by an experienced radiologist who was blinded to the clinical and pharmacological characteristics and randomization of the patients. The measurement time was 2–5 min, and the measurement error in testing the accuracy of the manikin was ≤3%.

The World Health Organization (WHO) criterion for osteoporosis is based on the measurement of BMD. According to the WHO diagnostic criteria^40,41^, the operational definition of osteoporosis in men was given as a BMD that was greater than 2.5 standard deviations (SD) below the mean value of young, healthy men, ie, a *T*-score < −2.5 SD. A *T*-score between 1.0 SD and −1.0 SD is considered normal or healthy, and a *T*-score between −2.5 SD and −1.0 SD indicates that you have low bone mass, although not low enough to be diagnosed with osteoporosis. The greater the negative number, the more severe the osteoporosis. We have previously measured the BMD of 12,159 normal individuals to obtain mean BMD values for different age and sex groups and obtained a mean peak BMD of 0.5340 g/cm^2^ for men. We then converted the range of peak BMD at each level into absolute values based on the *T*-score, i.e., absolute values of BMD greater than 0.467 g/cm^2^ is considered normal, less than 0.467 g/cm^2^ and greater than 0.364 g/cm^2^ is bone loss, and less than 0.364 g/cm^2^ is osteoporosis.

### Balance ability measurements

The secondary outcome measures were the balance ability. The balance ability (static and dynamic) of patients with SZ was assessed by three raters using the Berg balance scale (BBS) in the two groups pre- and post-test, to evaluate the changes in balance ability. The BBS scale was first reported by Katherine Berg in 1989 and is now widely recognized as an informative way to evaluate balance ability in clinical practice^42^. It is a quantitative test that combines most components of postural control through 14 functional activities, including reaching, bending, transferring, and standing: sitting and transferring safely between chairs; standing with feet apart, feet together, standing on a single leg, and feet in the tandem; Romberg position with eyes open or closed and reaching and stooping down to pick something off the floor. Each item is scored on a 5-point scale ranging from 0 to 4. Zero indicates the lowest level of function, and 4 is the highest level of function. The total score for all items is 56. The BBS is reliable (both inter- and intratester) and has been validated for validity^43^. The Cronbach alpha in this study was 0.851. The lower the score on the scale, the worse the balance and the higher the risk of falling. A score of 40 or less indicates the probability of falling.

### Data analysis

Statistical analysis was performed in SPSS Statistics, version 22.0 (IBM). All statistical analyses were teo-sided, and the significance threshold was set at *p*-values lower than 0.05.

Considering that patients who dropped out at the beginning of treatment did not receive treatment, the last observation carried forward analysis was not performed for patients who dropped out in the first week of treatment in this study. Demographic characteristics and markers of BMD at baseline between the DMT group and the TAU group were compared using analysis of variance (ANOVA) or chi-square tests.

Then, repeated-measure multivariate analysis of variance (RM-MANOVA) was performed to compare the efficacy of the two treatment groups by assessing the main effects (group and time) and the group-by-time interactions on the primary endpoint variables. Using the RM-MANOCA, researchers can control for an increased risk of Type 1 error.

After the RM-MANOVA, separate repeated measure ANOVA (RM-ANOVA) tests were followed up with the tests of individual outcome variables if RM-MANOVA yielded significant results. In the RM-ANOVA model, time (baseline and 3-month follow-up) was added as within-effect, group (DMT vs. TAU) was added as between-effect, and BMD and balance ability over time were independent variables, respectively.

If a significant difference was observed in the interaction effect of group and time in separate RM-ANOVA, an analysis of covariance with the baseline scores as covariates were conducted to test for the group differences in a 12-week follow-up after treatment. If there was no significant interaction between the group and time, no further statistical analysis was carried out.

## Results

### Demographic and clinical symptoms at baseline

Table 1 showed that no statistical differences were observed in the baseline data between the DMT and TAU groups (all *p* > 0.05). The baseline demographic characteristics, BMD, balance ability, and lipid profiles of the two groups were well-matched.

**Table 1:** Comparisons of demographic characteristics and biomarkers between the DMT group and the control group ($\bar{x}$ ± *s*).

Variables	TAU (*n* = 29)	DMT (*n* = 29)	*t* or *X*^2^ (*p*-value)	Dropouts (*n* = 2)	Finished (*n* = 58)	*t* or *X*^2^ (*p*-value)
Age (ys)	55.3 ± 3.9	55.4 ± 3.6	0.31 (0.97)	55.5 ± 2.12	55.3 ± 3.7	0.01 (0.95)
DOI (ys)	34.7 ± 4.3	33.7 ± 4.9	0.75 (0.46)	33.5 ± 3.5	34.2 ± 4.6	0.20 (0.83)
CDD (ys)	7.6 ± 4.4	8.7 ± 6.6	0.71 (0.48)	5.0 ± 1.4	8.1 ± 5.6	0.79 (0.43)
Smokers, *n* (%)	17 (58.6)	16 (55.2)	0.1 (0.79)	1 (50.0)	33 (56.9)	0.00 (1.00)
Calcium	2.4 ± 0.8	2.4 ± 0.1	0.25 (0.81)	2.2 ± 0.1	2.4 ± 0.1	1.43 (0.16)
BMD	0.41 ± 0.03	0.42 ± 0.03	1.83 (0.07)	0.4 ± 0.01	0.4 ± 0.03	0.48 (0.63)
Berg total score	44.5 ± 6.3	43.1 ± 5.2	0.89 (0.38)	42.0 ± 0.0	43.8 ± 5.8	0.43 (0.67)
Static balance	32.5 ± 3.4	31.5 ± 3.2	1.05 (0.29)	31.0 ± 0.0	32.0 ± 3.3	0.42 (0.68)
Dynamic balance	12.2 ± 3.4	12.2 ± 3.2	0.04 (0.97)	11.0 ± 0.0	12.2 ± 3.3	0.51 (0.61)

*ys* years, *DOI* duration of illness, *CDD* current disease duration, *BMD* bone mineral density.

### Effects of DMT intervention on BMD

RM-ANOVA analysis showed significant interaction effects of time and group on BMD, Berg total score, and static and dynamic balance (all *p* < 0.05) (Table 2), suggesting a statistically significant improvement in the DMT group compared to the TAU group from baseline to week 12. There were no significant main effects of time or group on BMD and balance ability (all *p* > 0.05).

**Table 2:** Comparisons of bone mineral density biomarkers after treatment using repeated measures ANOVA analysis ($\bar{x}$ ± *s*).

Variables	Control group		DMT group			
	At baseline (*n* = 29)	At follow-up (*n* = 29)	At baseline (*n* = 29)	At follow-up (*n* = 29)	Group effect *F*(*p*)	Group*time interaction effect *F*(p)
Calcium	2.4 ± 0.8	2.4 ± 0.1	2.4 ± 0.1	2.4 ± 0.8	0.1 (0.72)	0.05 (0.83)
BMD	0.4 ± 0.03	0.4 ± 0.4	0.4 ± 0.02	0.5 ± 0.06	6.5 (0.01)^*^	8.9 (0.004)^**^
Berg total score	44.5 ± 6.3	44.8 ± 7.7	43.1 ± 5.2	50.4 ± 4.5	58.0 (<0.001)^**^	47.1 (<0.001)^**^
Static balance	12.2 ± 3.4	12.9 ± 3.4	12.2 ± 3.2	15.9 ± 2.4	5.67 (0.02)^*^	7.0 (0.01)^*^
Dynamic balance	32.5 ± 3.4	32.2 ± 5.0	31.5 ± 3.2	35.5 ± 7.9	43.9 (<0.001)^**^	20.8 (<0.001)^**^

^*^p < 0.05, ^**^p < 0.01.

Further analysis of the two groups showed that compared to baseline, BMD, the Berg total score, the static and dynamic balance scores increased significantly after treatment in the DMT group, while there was no change in the TAU group (*p* > 0.05). Treatment with DMT demonstrated a significant improvement in BMD from baseline to week 12 (0.03, 95% confidence intervals [CI]: 0.01–0.05). For the Berg total score and static and dynamic balance, the mean changes in the DMT group were 7.3 (95% CI: 5.6–9.0), 4.0 (95% CI: 0.9–7.1) and 3.7 (95% CI: 2.6–4.8), respectively.

### Potential predictors of treatment response

Multiple linear regression analyses were used to determine potential predictors of treatment response. Considering a strong positive association between age and duration of illness and to avoid including completely collinear covariates in the analysis, we chose age as a covariate in the regression model. The results showed that baseline BMD was a significant predictor of improvement from baseline to week 12 in the DMT group (*β* = 0.58, *t* = 5.2, *p* < 0.001, 95% CI: 0.69–1.57) (*R*^*2*^ = 0.35).

## Discussion

This study found that after 12 weeks of treatment, BMD, Berg total score, and static and dynamic balance increased significantly in the DMT intervention group compared with the TAU group in hospitalized male veterans with SZ.

Osteoporosis is a metabolic condition characterized by decreasing bone mass and destruction of the microarchitecture of bone tissue, leading to increased bone fragility and the risk of fracture^44^. Current studies on the treatment of osteoporosis have mainly focused on menopausal women and older people, and few therapeutic interventions have been reported for high-risk populations of long-term hospitalized SZ patients with osteoporosis. Although there are several studies on falls in the general population, to our best knowledge, this is the first study to investigate the efficacy of a DMT intervention on BMD in long-term veterans with SZ. Moreover, our study also specialized in that, unlike the patients recruited in other studies, we minimized the effect of age on the results by enrolling only hospitalized veterans aged 40–60 years. On the other hand, we also took into account the low incidence of low BMD and osteoporosis in individuals under 40 years of age and the increased risk of falls during DMT intervention in those over 60 years of age.

In the present clinical study, we showed that the DMT intervention was effective to increase BMD and prevent falls in long-term hospitalized male veterans with SZ. Moreover, we found that the Berg total score, dynamic balance, and static balance scores were significantly increased in the DMT group, but no changes in the TAU group. Numerous studies have found that long-term use of antipsychotic drugs leads to abnormalities in biomarkers of lipid metabolism and bone turnover, which can negatively affect bone metabolism in patients with SZ^45–48^. In clinical practice, patients with osteoporosis usually improve significantly with medication and physical activity^49^. However, there are multiple difficulties in the treatment of osteoporosis comorbidity in veterans with mental disorders who have been hospitalized for a long period. Long-term hospitalization, antipsychotic medications, reduced outdoor activity and exercise, lack of regular bone density testing and calcium supplements in hospitalized patients, and a significant reduction in the duration and frequency of outdoor activity over the last three years due to the COVID-19 epidemic, have contributed to an increased risk of fractures from falls in long-term hospitalized veterans with SZ.

Notably, our findings demonstrated that DMT intervention helped to improve the balance function of long-term hospitalized patients, especially the dynamic balance function. Dynamic balance plays a crucial role before postural changes lead to a fall. Individuals with SZ who do not keep physically active or fit tend to have poorer balance, which increases the likelihood of falling. DMT intervention uses dance and movement to further the emotional, cognitive, physical, spiritual, and social integration of the individual in a non-verbal way. Through simple movement exercises and playful interaction, DMT is particularly applicable for improving the balance ability in long-term hospitalized veterans with SZ. Our findings suggest that early implementation of continuous DMT interventions could reduce or delay the onset of bone loss, increase balance ability, and decrease the risk of fall fractures and the severity of fall injuries of long-term hospitalized veterans with SZ. Thus, this clinical trial provides further evidence for the DMT intervention for patients with SZ.

Several limitations should be noted in this study. First, the sample size was small for a clinical trial to examine the efficacy of DMT intervention on BMD and falls in patients with SZ. Thus, we were unable to investigate the effects of sex, age, different types of antipsychotics, duration of antipsychotic exposure, and other fracture risk factors on the outcomes. Second, this study was not a double-blind design. The raters did not know which group the patients had been assigned to during the trial. However, the patients knew which group they were assigned to since they often revealed their treatment groupings when they shared their experiences. Third, it would be better to use the risk of falls, the risk of fractures, the risk ratio for disability, and quality of life as the main outcomes, however, this study only investigated the effect of short-term treatment on the balance ability. Short-term treatment may have little impact on the outcomes such as the risk of falls, the risk ratio for disability, and quality of life. Forth, although the efficacy of DMT on lower limb muscle strength and balance is an important point, which are significant predictors of fractures and falls, the improvements in cognitive functions, social interaction, and depressive symptoms from the intervention also need to be assessed in future studies. Fifth, all participants were male inpatients with SZ and our findings may not generalize to female patients.

In conclusion, falling is one of the serious accidents in psychiatric hospitals, and veterans with SZ are at increased risk of falls and fractures due to physical inactivity caused by long-term hospitalization and the use of antipsychotic drugs, combined with decreased BMD and balance ability. DMT interventions can increase bone mass and balance ability in patients with SZ, thereby reducing the risk of fracture in case of falls and further reducing the stress of clinical care. Our study suggests that DMT intervention is a worthwhile therapy for long-term hospitalized veterans with SZ in clinical practice.

## Data and materials availability

The datasets generated and analyzed during the current study are available from the corresponding author on reasonable request.

## Supplementary information

supplementary file
