# Supplementary material for: Dance/movement therapy for improving balance ability and bone mineral density in long-term patients with schizophrenia: a randomized controlled trial
Source: Schizophrenia (Heidelb). 2023 Jul 31;9(1):47. doi: 10.1038/s41537-023-00373-w (PMC10390548; doi:10.1038/s41537-023-00373-w)
Supplement: Supplementary file 1 — supplementary file [file 41537_2023_373_MOESM1_ESM.docx]

***Supplementary file***

The detailed process of DMT intervention is divided into four stages.

Stage 1: Relationship building phase (5-10 minutes) - Familiarizing oneself with the environment and relaxing the mind and body. The therapist asks the patient to freely walk around the room, get acquainted with the surroundings, and feel safe and receptive. The therapist also conducts an initial analysis and evaluation of the patient's body posture and physical condition through simple warm-up activities.

Stage 2: Adaptation phase (10-15 minutes) - Awaking the body and enhancing self-awareness. The therapist gently guides the patient to move and stretch their body while introducing themselves through various movements, rhythms, breathing sounds, and other means. The therapist observes the patient closely and responds to their movements, while guiding them to express and listen attentively.

Stage 3: Rehabilitation phase (30 minutes) - Improving initiation and exploring creative movement and sharing physical and emotional feelings. This stage is divided into two parts: 1) Free movement (20 minutes) - The therapist plays different types of music, either dynamic or lyrical, and guides the patient to express their inner state through body movements without deliberately learning any specific moves. They just need to feel and dance freely with the rhythm, while being guided on how to maintain body balance and stable breathing during posture changes. The therapist not only guides the release of emotions, the safety of dance movements, and the adjustment of breathing frequency but also observes the patient's physical ability and provides care in a timely manner to those who are not physically fit, allowing them to take a rest or complete some movements while sitting, ensuring patient safety. 2) Sharing and communication (10 minutes) - The therapist guides the patient to share their dance experience in various ways, either through verbal expression or by using writing and drawing.

Stage 4: Closing phase (10min): Review the treatment and end positively using the principle of combining body movements and conversation. The patient is guided through physical relaxation exercises to adjust the body and mind. The therapist gives a short review of the activity to organize, comment and encourage. Finally, patients are invited to form a circle to express their blessings and gratitude, ending in a positive situation.
